# Sensorimotor Rhythm-Brain Computer Interface With Audio-Cue, Motor Observation and Multisensory Feedback for Upper-Limb Stroke Rehabilitation: A Controlled Study

**DOI:** 10.3389/fnins.2022.808830

**Published:** 2022-03-11

**Authors:** Xin Li, Lu Wang, Si Miao, Zan Yue, Zhiming Tang, Liujie Su, Yadan Zheng, Xiangzhen Wu, Shan Wang, Jing Wang, Zulin Dou

**Affiliations:** ^1^Department of Rehabilitation Medicine, The Third Affiliated Hospital of Sun Yat-sen University, Guangzhou, China; ^2^Institute of Robotics and Intelligent Systems, School of Mechanical Engineering, Xi’an Jiaotong University, Xi’an, China; ^3^Department of Rehabilitation Medicine, Shenzhen Hengsheng Hospital, Shenzhen, China; ^4^Air Force Medical Center, PLA, Beijing, China

**Keywords:** stroke, motor imagery, brain computer interface, mu rhythm, rehabilitation

## Abstract

Several studies have shown the positive clinical effect of brain computer interface (BCI) training for stroke rehabilitation. This study investigated the efficacy of the sensorimotor rhythm (SMR)-based BCI with audio-cue, motor observation and multisensory feedback for post-stroke rehabilitation. Furthermore, we discussed the interaction between training intensity and training duration in BCI training. Twenty-four stroke patients with severe upper limb (UL) motor deficits were randomly assigned to two groups: 2-week SMR-BCI training combined with conventional treatment (BCI Group, BG, *n* = 12) and 2-week conventional treatment without SMR-BCI intervention (Control Group, CG, *n* = 12). Motor function was measured using clinical measurement scales, including Fugl-Meyer Assessment-Upper Extremities (FMA-UE; primary outcome measure), Wolf Motor Functional Test (WMFT), and Modified Barthel Index (MBI), at baseline (Week 0), post-intervention (Week 2), and follow-up week (Week 4). EEG data from patients allocated to the BG was recorded at Week 0 and Week 2 and quantified by mu suppression means event-related desynchronization (ERD) in mu rhythm (8–12 Hz). All functional assessment scores (FMA-UE, WMFT, and MBI) significantly improved at Week 2 for both groups (*p* < 0.05). The BG had significantly higher FMA-UE and WMFT improvement at Week 4 compared to the CG. The mu suppression of bilateral hemisphere both had a positive trend with the motor function scores at Week 2. This study proposes a new effective SMR-BCI system and demonstrates that the SMR-BCI training with audio-cue, motor observation and multisensory feedback, together with conventional therapy may promote long-lasting UL motor improvement.

**Clinical Trial Registration:** [http://www.chictr.org.cn], identifier [ChiCTR2000041119].

## Introduction

Stroke is a leading cause of mortality and disability worldwide (Johnson et al., 2019; Zhou et al., 2019). Up to 66% of stroke survivors experience upper limb (UL) motor impairments, which result in functional limitations in activities of daily living and decreased life quality (Kwah et al., 2013; Morris et al., 2013).

Electroencephalography (EEG)-based sensorimotor rhythm (SMR) brain computer interface (BCI) is a novel technology that can enhance activity-dependent neuroplasticity and restore motor function for stroke survivors (Ang et al., 2014a; Lazarou et al., 2018; Jeunet et al., 2019). SMRs can be measured over the sensorimotor cortex and modulated by actual movement, motor intention, or motor imagery (MI; Frenkel-Toledo et al., 2014; Yuan and He, 2014). Task-related modulation in EEG-based SMRs is usually manifested as event-related desynchronization (ERD) or event-related synchronization (ERS) in low-frequency components [mu rhythm (8–12 Hz) and beta rhythm (13–26 Hz)] (Pfurtscheller and Lopes da Silva, 1999), which forms the basis of neural control in EEG-based SMR-BCI (Yuan and He, 2014). Furthermore, patients with stroke or spinal cord lesions can control physical or virtual devices via SMR-BCI (Prasad et al., 2010; Caria et al., 2011; Ang et al., 2014a,b; Dodakian et al., 2014; McCrimmon et al., 2014; Ono et al., 2014; Yuan and He, 2014; Ang and Guan, 2015; Bartur et al., 2015; Pichiorri et al., 2015; Zich et al., 2015; Shu et al., 2017, 2018; Barsotti et al., 2018; Biasiucci et al., 2018; Lazarou et al., 2018; Lee et al., 2018; Norman et al., 2018; Jeunet et al., 2019; Song and Kim, 2019; Chen et al., 2020; Foong et al., 2020), which raises the possibility of SMR-BCI training for stroke rehabilitation.

Several clinical studies have investigated the effect of SMR-BCI systems and demonstrated the significantly positive outcomes on motor function improvement for stroke patients (Ang and Guan, 2015). Ramos-Murguialday et al. (2013) and Ang et al. (2014a,b) stated the BCI training had better efficacy than sham-BCI for stroke rehabilitation. Besides, Cantillo-Negrete et al. (2021) investigated the clinical and physiological effects of SMR-BCI intervention and conventional therapy for upper limb stroke rehabilitation and a revealed similar positive impact of the two therapy methods. Thus, SMR-BCI training, together with conventional therapy, is a suitable therapy option for stroke recovery.

To improve the efficacy of SMR-BCI, various SMR-BCI systems combined with sensory stimulation, motor observation (MO) have been proposed. Shu et al. (2017, 2018) and Ren et al. (2020) improved the SMR-BCI performance via proprioceptive stimulation before the motor imagery (MI) task. Choi et al. (2019), Nagai and Tanaka (2019), and Fujiwara et al. (2021) found users’ ERD/ERS was enhanced when they performed MI task with motor observation. It is recognized that enhanced ERD/ERS of stroke patients, meaning enhanced motor-related cortical activation (Pfurtscheller and Lopes da Silva, 1999; Pfurtscheller et al., 2006b), can improve users’ engagement and decoding accuracy for BCI system, which could help maximize brain plasticity and restore motor and cognitive function for stroke patients (Bundy et al., 2017; Nagai and Tanaka, 2019). Furthermore, Velasco-Álvarez et al. (2013) designed an audio-cued SMR-BCI system and showed its availability.

Besides, various neuro-feedback has been added to make SMR-BCI system a closed loop for better effect on stroke recovery. Ramos-Murguialday et al. (2013) and Ang et al. (2014a,b, 2015) demonstrated that SMR-BCI with robotic feedback was the most popular feedback method and had positive efficacy for stroke rehabilitation. Pichiorri et al. (2015) and Foong et al. (2020) observed that SMR-BCI with visual feedback showed its excellence for stroke recovery. Auditory feedback may also improve SMR-BCI performance (Nijboer et al., 2008; McCreadie et al., 2013, 2014). Several researchers found the users’ ERD/ERS was improved via SMR-BCI with proprioceptive feedback (Vukelić and Gharabaghi, 2015; Barsotti et al., 2018).

For stroke patients, the ability to keep attention is weakened due to of brain damage. To enhance the ERD/ERS and maximize the efficacy of BCI training, we propose a new SMR-BCI system with audio-cue, MO, and multisensory (auditory, visual, and robotic) feedback and investigate the effectiveness of this system.

Another urgent investigation, which should be further explored, is optimal and safe exercise prescription (e.g., training intensity and duration) (Farrell et al., 2020; Luo et al., 2020). We used the definition of training intensity and duration in a review (Antje et al., 2020) as a reference: (1) training intensity (high: five times per week vs. moderate: 2–3 times per week), (2) training duration (short: 2–3 weeks vs. long: 4–8 weeks). Most of the SMR-BCI intervention proposed fell into the pattern of moderate training intensity with long training duration, involving 10 sessions (twice a week) (McCrimmon et al., 2014), 12 sessions (three times a week) (Ang et al., 2014a; Pichiorri et al., 2015; Chen et al., 2020), 18 sessions (three times a week) (Foong et al., 2020), 20 sessions (daily training exclude weekends) (Ramos-Murguialday et al., 2013; Wu et al., 2020) and 24 sessions (twice a week) (Sebastián-Romagosa et al., 2020), which have shown positive effects on stroke rehabilitation. Few studies have addressed the pattern of high training intensity with short training duration. One clinical trial involved 10 training sessions, but each session of BCI training lasted up to 40 min (Frolov et al., 2017). As our group suggests, motor function recovery and the brain networks of stroke patients could be improved significantly by 4-week SMR-BCI intervention combined with convention training compared to only conventional treatment (Wu et al., 2020), which leads us to ponder whether a high training intensity with short duration SMR-BCI intervention will get better influence. If that works, stroke patients will restore the ability to live independently faster.

As mentioned above, there are two purposes of this study. Firstly, to investigate the efficacy of non-invasive EEG-based SMR-BCI with audio-cue, MO, and multisensory (robotic, visual, and auditory) feedback, together with conventional therapy, for upper limb rehabilitation of stroke patients. Secondly, to discuss the influence of stroke rehabilitation after a high training intensity with short duration SMR-BCI intervention.

## Materials and Methods

### Subjects

All patients were recruited from The Third Affiliated Hospital of Sun Yat-sen University.

The following inclusion criteria were applied: (1) age between 18 and 75 years; (2) hemiparesis resulting from a unilateral brain lesion, as confirmed by magnetic resonance imaging (MRI), with a time since stroke (TSS) of 6–24 weeks before study enrollment; (3) moderate to severe hand paralysis, as determined by a Brunnstrom score <IV; (4) sufficient cognition to follow simple instructions and understand the purpose of the study (Susanto et al., 2015).

The exclusion criteria were: (1) recurrent stroke; (2) other neurological, neuromuscular, orthopedic diseases; (3) shoulder or arm contracture/pain; (4) severe aphasia, dementia, psychotic symptoms, or a scalp deformity due to surgery, or those who could not undergo EEG recording for other reasons, such as involuntary movements; or (5) receiving other clinical central nervous system interventions (Susanto et al., 2015).

Based on experience, EEG acquisition devices used on women got poor signals because of their long hair, so we tried to select male patients.

**FIGURE 1:**
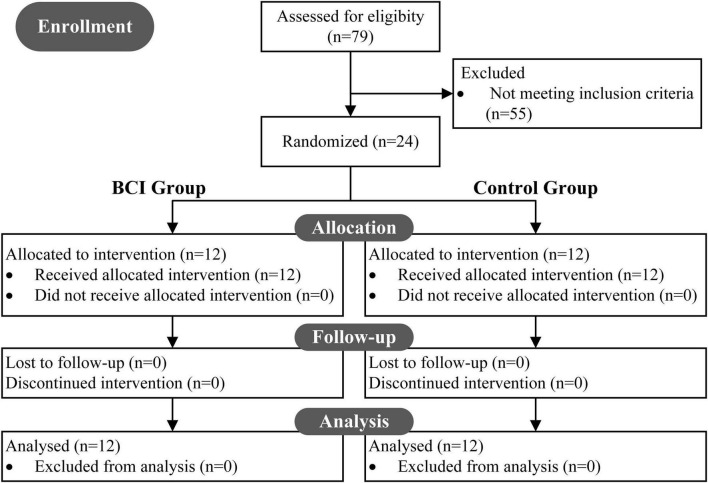
CONSORT diagram: a flow from recruitment through follow-up and analysis. In all, 79 patients were screened to be eligible for the study, and 55 were excluded. 24 underwent intervention and were randomly assigned to two groups (BCI group, BG; control group, CG). All 24 recruited patients received follow-up analysis.

From 79 potentially eligible patients, 24 stroke survivors were allocated to the intervention and received follow-up analysis (see Figure 1). Twenty-four patients were randomly assigned into one of the two groups: (1) the BCI therapy group (BCI Group, BG), and (2) the non-BCI group (Control Group, CG).

### Study Design

All subjects were recruited to receive a total of 10 training sessions, lasting for 3 h per day, 5 days per week (excluding weekends). Each training session consisted of 1-h BCI therapy and 2-h conventional treatment for the BG, while only 3-h conventional treatment for the CG.

Each SMR-BCI intervention consisted of one calibration session and about ten BCI training sessions (1 h). Conventional treatment included physiotherapy and occupational therapy involving shoulder, elbow and hand training: neuromuscular electrical stimulation, passive joint activity, strength training, stretch and Activities of Daily Living (ADL) training. Specifically, for the patients who belonged to the BG, the hand training part in conventional treatment was excluded.

The clinical measure scales were measured at three time points: at baseline (Week 0), at post-intervention (Week 2), and at follow-up week (Week 4). Notably, the patients in both groups were still hospitalized after 2 weeks of intervention and received conventional therapy.

### Sensorimotor Rhythm-Brain Computer Interface System Description

**FIGURE 2:**
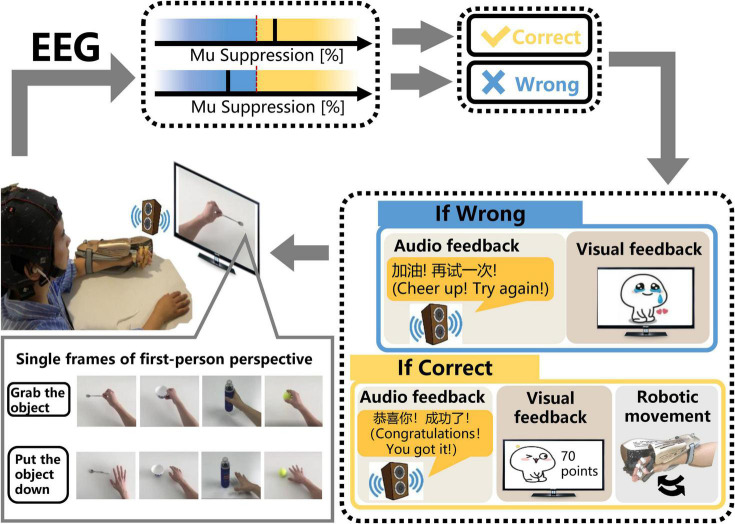
The schematic diagram of the SMR-BCI system. According to audio-cue, subjects imagine “grabbing the object” or “putting the object down” accompanied by observing the video. BCI system calculates the mu suppression of subjects’ EEG data and recognizes patients’ intention via comparing the mu suppression value with the threshold. If the purpose is identified correctly, the system will give multisensory (robotic, auditory, and visual) feedback. On the contrary, the system will still provide corresponding auditory and visual feedback, but the robot will maintain the previous state.

The SMR-BCI system is shown in Figure 2. Patients wore EEG caps with 16 active electrodes (g.Nautilus, g.tec medical engineering GmbH, Austria) and the affected hand wore an exoskeleton hand robot (RHB-III. Shenzhen Rehab Medical Technology Co., Ltd., China). The patients were advised to avoid blinking, coughing, chewing and minimize any body movements when performing tasks.

After undergoing a calibration session, including only one trial (described in the section “Sensorimotor Rhythm-Brain Computer Interface Session”), subjects performed MI training sessions according to the audio-cue and observed the corresponding video on the monitor. If the mu suppression was detected in the motor intention classification area (yellow shading), the exoskeleton hand would assist the paretic hand in grasping or opening action according to the MI task cued on the video. Once the robot was triggered, it would complete the movement regardless of the mu suppression during the motion. Then, the system provided audiovisual feedback with a training score (see Figure 2). In contrast, the robot would maintain the previous state and the BCI system would give corresponding audiovisual feedback (see Figure 2) if the mu suppression didn’t reach the threshold (60%) and was in the rest area (blue area) within 12 s. During the time of performing MI task, the BCI system would detect the mu suppression of the subject three times, each time lasting 4 s. If the mu suppression was tested above the threshold in the first 4 s, the feedback meaning “successful” would be given. If not, the BCI system would monitor the mu suppression for the next 4 s, and so on, until 12 s.

The mu suppression (Oberman et al., 2008; Sun et al., 2017) of EEG SMR recorded by electrodes was used for a brain-controlled switch. EEG acquisition and processing details are described in section “Electroencephalography Acquisition, Processing, and Analysis.”

### Sensorimotor Rhythm-Brain Computer Interface Session

**FIGURE 3:**
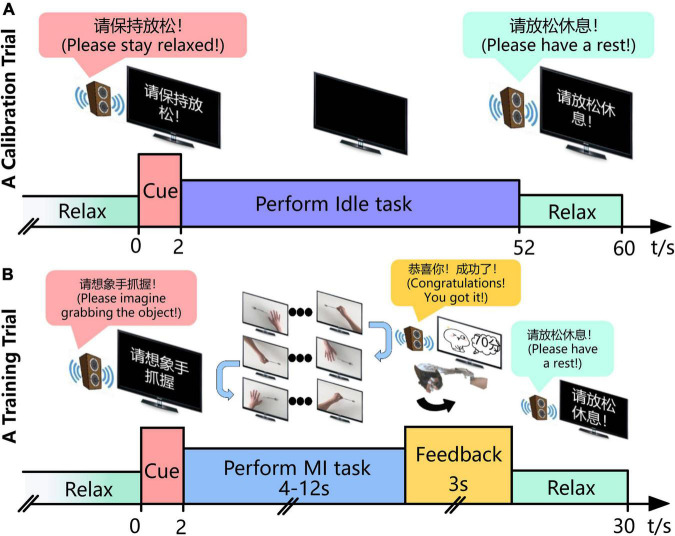
Experimental time course of the BCI intervention for stroke patients**’** rehabilitation. **(A)** In the calibration trial, the patient gets an audio cue to keep still and eyes closed for 50 s and the EEG when performing the “Idle task” is collected. **(B)** In the training trial, the subject imagines, for instance, “grabbing the objects” according to the audio-cue and monitor from the first-person perspective, third-person perspective, or inverse first-person perspective in turn until his/her intention is recognized. Then, the system gives the corresponding auditory, visual and/or robotic feedback during the following 3 s. The MI task of “grabbing the object” and “putting the thing down” is cued alternatively if the intention is identified successfully, or it maintains the previous one.

Before conducting MI sessions, patients first performed a calibration trial, to get the “Idle-state Potential,” where subjects were instructed to keep still at resting state with eyes closed, called “idle task.” A calibration trial lasted about 60 s. Figure 3A shows the timing of a calibration trial.

Each MI training session completed approximately 10 runs, each consisting of 10 trials. A break of 3–5 min was given after each run. Figure 3B shows the timing of a training trial. Each trial lasted 30 s. Subjects performed the MI task according to audio-cue and the video on the monitor. In addition, the video of three perspectives (first-person perspective, third-person perspective, and inverse first-person perspective) was played in turn. Once the mu suppression of the patient exceeded the threshold, the system would give the corresponding robotic, auditory, and visual feedback.

### Electroencephalography Acquisition, Processing, and Analysis

Electroencephalography was recorded using g.Nautilus headset (g.tec medical engineering GmbH, Austria), which provided 16 active electrodes placed in the international 10–20 system positioning: FP1, FP2, FC3, FZ, FC4, C1, C2, CZ, C3, C4, CP3, PZ, CP4, PO7, PO8, and POz. The reference electrode and ground electrode were located on the right mastoid and left mastoid, respectively. Impedances for all electrodes were maintained at <5 kΩ throughout the experiment. Raw EEG recordings were sampled at 256 Hz. Signals were also processed in real-time by the amplifier using an analog bandpass filter (0.5–60 Hz) and a notch filter (48–52 Hz) to remove artifacts and power line interference.

After preprocessing, the EEG of C3/C4 electrodes covered over the primary motor cortex was used for BCI control. The signals were processed by a bandpass filtered (4th order Butterworth filter) between mu rhythm (8–12 Hz) with a Hamming window. Mu suppression reflects an ERD of the EEG caused by an increase in neural activity (Sun et al., 2017), which is used for the value of recognition in this BCI system. The mu suppression score was calculated according to the following equation (Braadbaart et al., 2013):


(1) $$muSupp\;=-\frac{m\,u\,P_{t\,a\,s\,k}-m\,u\,P_{i\,d\,l\,e}}{m\,u\,P_{i\,d\,l\,e}}\;\times 100\%$$

where *muSupp* represents the mu rhythm suppression value, *muP*_*task*_ represents the mu rhythm power of EEG while performing “MI task,” and *muP*_*idle*_ is the mu rhythm power of EEG while performing “Idle task.”

In the offline analysis, the EEG recorded by channel C3 and channel C4 was analyzed. The segments containing gross artifacts (identified by visual inspection) were excluded for further analysis. These EEG processes were carried out in MATLAB (The MathWorks, Inc., Natick, MA, United States).

Firstly, we analyzed the mu suppression as the equation (1) and its correlation with motor function scores. The following method was used to compute the mu suppression value:

1. Bandpass filtering of 8–12 Hz on the EEG recorded during 2-week SMR-BCI training session. While the EEG on time segment 5–45 s (see Figure 3A) was analyzed for idle-state, the EEG when performing MI tasks at Week 0 and Week 2 (see Figure 3B) was investigated for MI-state.
2. Squaring the bandpass-filtered samples to obtain power samples.
3. Computing the power value when performing “Idle task” by averaging the idle-state power samples.
4. Computing the power value when performing “MI task” by averaging the MI-state power samples in one session.
5. Computing the mu suppression using the equation (1).

### Outcome Measures

Fugl-Meyer Assessment-Upper Extremities (FMA-UE) assessment scale was used as the primary motor function outcome measure because of its high reliability. In this study, FMA-UE only referred to the upper extremity motor function part with a total score of 66 (Fuglmeyer et al., 1975; Page et al., 2012).

Secondary outcome measures were the Wolf Motor Function Test (WMFT; Wolf et al., 2001) and the Modified Barthel Index (MBI; Mahorney, 1965). The WMFT consists of 15 tasks (six joint-segment tasks, nine functional tasks; maximum score = 75), each of which should be performed within 120 s. The MBI is used to measure performance in ADL.

Mean change of FMA-UE and WMFT scores were compared with its estimated minimal clinically significant difference (MCID) values (Der Lee et al., 2001; Page et al., 2012) and estimated minimal detectable change (MDC) value (Lin et al., 2009), respectively.

### Statistical Method

All demographic and clinical data were analyzed using SPSS version 23.0 (IBM Inc., Chicago, IL, United States). The variables tested normal (using Shapiro–Wilk test) were expressed as the mean ± standard deviation, and the two-tailed unpaired *t*-test was used for intergroup comparison while the two-tailed paired *t*-test was for intragroup comparison. Non-normally distributed data were expressed as the median with 25 and 75% quartile, and the Mann–Whitney *U* test was applied for intergroup comparison while the Wilcoxon ranked sum test was for intragroup comparison.

Two-way repeated measures ANOVA was performed for the functional scale scores (FMA-UE, WMFT, and MBI) with time (Week 0, Week 2, and Week 4) as the within-subject factor.

Associations between the clinical scores (at Week 2) and the mu suppression (at Week 2) were assessed using Pearson’s (if the two variables are both normally distributed), or Spearman’s correlation. Statistical significance was set at *p* < 0.05 for all analyses.

## Results

### Demographics

The demographic information of the patients (at baseline) is shown in Table 1. For demographic information, standard variables, including age and clinical scale scores, were analyzed by the parametric tests while TSS was analyzed by the non-parametric test. The chi-square test was used to identify difference in rates among the groups.

**TABLE 1:** Demographic information of the patients.

Characteristic	Control group (*n* = 12)	BCI group (*n* = 12)	*t/Z/χ^2^*	*P*
Age (years)	55.0 ± 12.2	43.8 ± 14.7	−2.040^b^	0.054
Gender (male: female)	12:0	12:0		
Affected hand (right: left)	8:4	9:3	0.202^a^	0.653
Stroke type (isch: hemo)	9:3	7:5	0.750^a^	0.386
TSS (month)	4.3 ± 2.6	4.0 (2.0, 11.3)	−0.555^c^	0.579
STROKE PERIOD, N				
Subacute (1–6 months from onset)	10	7		
Chronic (>6 months from onset)	2	5		
LESION LOCALIZATION, N				
Cortical	4	3	0.202^a^	0.653
Subcortical	8	9		
MEASUREMENTS (baseline)				
FMA-UE	24.3 ± 17.1	22.6 ± 13.7	−0.264^b^	0.795
WMFT	27.8 ± 19.8	29.1 ± 16.6	0.179^b^	0.859
MBI	47.0 ± 32.9	57.9 ± 28.9	0.863^b^	0.397

*hemo, hemorrhagic stroke; isch, ischemic stroke; TSS, time since Stroke onset; N, number; FMA-UE, Fugl-Meyer Assessment-Upper Extremities; WMFT, Wolf Motor Functional Test; MBI, Modified Barthel Index. ^a^Chi-square test. ^b^two-tailed unpaired t-test. ^c^Mann–Whitney U test.*

There were no significant demographic differences in age (two-tailed unpaired *t*-test, *p* = 0.054), sex (all males), and affected hand (chi-square test, *p* = 0.653). Similarly, there were no significant differences in stroke type (chi-square test, *p* = 0.386), and TSS (Mann–Whitney *U* test, *p* = 0.579). In addition, patients in the two groups had similar levels of baseline clinical scores including FMA-UE (two-tailed unpaired *t*-test, *p* = 0.795), WMFT (two-tailed unpaired *t*-test, *p* = 0.859) and MBI (two-tailed unpaired *t*-test, *p* = 0.397).

### Efficacy Measurements

Two-way repeated-measures ANOVA (FMA-UE, WMFT, and MBI, by Week 4, with time as the within-subjects factor and group as the between-subjects factor) showed a significant time × group interaction on FMA-UE (*F* = 18.629, *p* < 0.01) and WMFT (*F* = 10.252, *p* = 0.001) and no significant time × group interaction on MBI (*F* = 0.500, *p* = 0.613). The results showed that time had a significant effect on FMA-UE (*F* = 120.626, *p* < 0.01), WMFT (*F* = 121.760, *p* < 0.01), and MBI (*F* = 17.228, *p* < 0.01), but no significant effect for group on FMA-UE (*F* = 0.005, *p* = 0.947), WMFT (*F* = 0.180, *p* = 0.675), and MBI (*F* = 1.614, *p* = 0.217).

Fugl-Meyer Assessment-Upper Extremities, WMFT, and MBI scores of the BG and that of the CG were significantly improved in Week 2 and Week 4. The results are shown in the Table 2. No significant differences were found between groups for clinical scores at any measurement point.

**TABLE 2:** Efficacy measures by FMA-UE, WMFT, and MBI for BG and CG.

Outcome measures	Mean ± SD			*p*-value		
	Week 0	Week 2	Week 4	Week 0 vs. Week 2	Week 0 vs. Week 4	Week 2 vs. Week 4
**BCI group**						
FMA-UE	22.58 ± 13.71	27.67 ± 15.99	35.75 ± 14.26	0.003**	<0.001**	<0.001**
WMFT	29.08 ± 16.58	36.58 ± 18.35	44.50 ± 16.89	0.001**	<0.001**	<0.001**
MBI	57.92 ± 28.94	71.25 ± 20.53	76.50 ± 20.26	0.004**	0.001**	<0.001**
**Control group**						
FMA-UE	24.25 ± 17.08	28.92 ± 18.14	31.50 ± 17.79	0.002**	<0.001**	<0.001**
WMFT	27.75 ± 19.75	34.17 ± 22.90	38.08 ± 22.92	0.001**	<0.001**	<0.001**
MBI	47.00 ± 32.87	55.92 ± 29.18	61.33 ± 29.67	0.007**	0.003**	0.026*

Measures with statistically significant (p < 0.05) changes are indicated with an *, p < 0.01 changes are indicated with an **.

As shown in Table 3, overall improvements of outcome measure scores of the BG were higher than that of the CG, and BG had significant improvement differences in FMA-UE and WMFT changes at Week 4 (Week 0-based change) compared to the CG.

**TABLE 3:** Intergroup comparison for outcome measure scores improvements.

Improvement		Control group (*n* = 12)	BCI group (*n* = 12)	*t/Z/χ^2^*	*P*
Week 2−Week 0	FMA-UE	4.7 ± 3.9	4.5 (2.0, 5.3)	−0.118^c^	0.906
	WMFT	6.0 (2.8, 7.3)	7.5 ± 5.5	−0.695^c^	0.487
	MBI	6.5 (4.8, 8.5)	9.0 (5.5, 16.8)	−0.955^c^	0.339
Week 4−Week 0	FMA-UE	6.0 (5.8, 7.5)	13.2 ± 3.0	−3.135^c^	0.002
	WMFT	10.3 ± 5.5	15.4 ± 5.1	2.346^b^	0.028
	MBI	10.0 (8.0, 13.8)	18.6 ± 14.6	−1.187^c^	0.235

*FMA-UE, Fugl-Meyer Assessment-Upper Extremities; WMFT, Wolf Motor Functional Test; MBI, Modified Barthel Index. ^b^two-tailed unpaired t-test. ^c^Mann–Whitney U test.*

**FIGURE 4:**
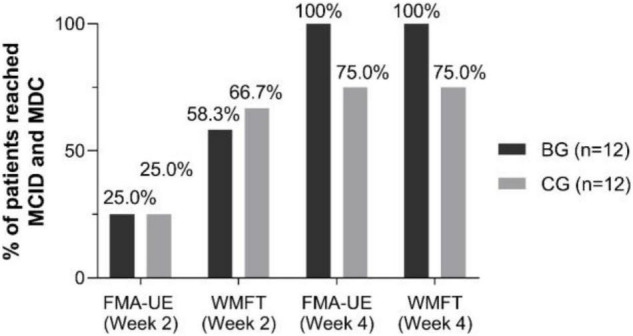
Percent of patients reached minimal clinically important difference (MCID) and minimal detectable change (MDC) by FMA-UE and WMFT scores in each group at Week 2 and Week 4.

Importantly, 5.25 points and 5.55 points have been estimated to represent the minimal clinically significant difference (MCID) of FMA-UE (Cervera et al., 2018) and minimal detectable change (MDC) of WMFT (Susanto et al., 2015), respectively. At Week 4, the increase of FMA-UE and WMFT surpassed MCID and MDC for all the patients in the BG (Figure 4).

### Electroencephalography Results

**FIGURE 5:**
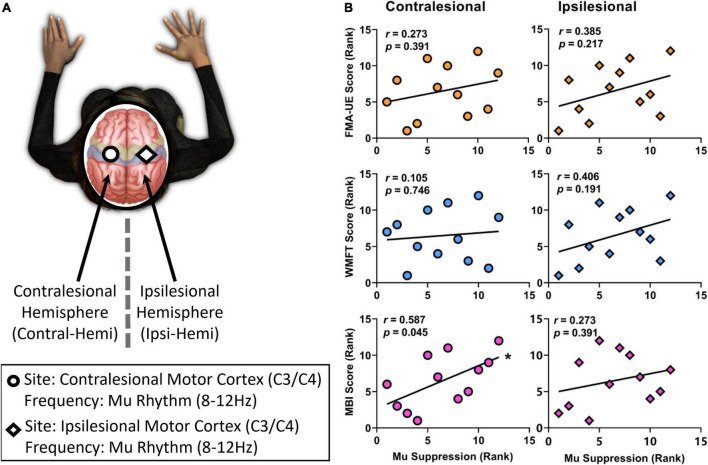
The correlation between the ranked mu suppression value and the ranked clinical scale scores (FMA-UE, WMFT, MBI scores). **(A)** Analyses were performed using EEG activity at the frequency used for BCI control but an electrode in the contralesional hemisphere, at the frequency used for BCI control but an electrode in the ipsilesional hemisphere. **(B)** While there was a significantly positive correlation between mu suppression of contralesional hemisphere and MBI scores after 2-week BCI intervention, others showed no significantly positive trend. Significant (*p* < 0.05) changes are indicated with an *.

The mu suppression values of bilateral cortex were compared before and after MI-BCI training (Figure 5A). No significant change in the bilateral hemisphere was found after BCI training [Ipsilesional hemisphere, *muSupp*_*Week0*_ = 45.7735 ± 28.0009, *muSupp*_*Week2*_ = 56.8294 (47.9067, 60.6983), Wilcoxon ranked sum test, *p* = 0.875; Contralesional hemisphere, *muSupp*_*Week0*_ = 62.4475 (21.4197, 72.9325), *muSupp*_*Week2*_ = 53.8142 ± 25.9915, Wilcoxon ranked sum test, *p* = 0.388]. Also, there was no significant difference in mu suppression between hemispheres (Week 0: Mann–Whitney *U* test, *p* = 0.670; Week 2: Mann–Whitney *U* test, *p* = 0.768).

Correlation analysis was performed to assess the relationship between the ranked mu suppression (Week 2) and the ranked clinical scale scores (Week 2) in the BG (Figure 5B). After 2-week comprehensive rehabilitation, including BCI and conventional interventions, the mu suppression of contralesional hemisphere had a significantly positive correlation with MBI scores (Pearson *r* = 0.587, *p* = 0.045). While there was no significant correlation between mu suppression of contralesional hemisphere and FMA-UE (Pearson *r* = 0.273, *p* = 0.391), or WMFT (Pearson *r* = 0.105, *p* = 0.746), they had a positive correlation trend. On the ipsilesional motor cortex, there was also a positive correlation trend between mu suppression and motor function (FMA-UE: Pearson *r* = 0.385, *p* = 0.217; WMFT: Pearson *r* = 0.406, *p* = 0.191; MBI: Pearson *r* = 0.273, *p* = 0.391). Pearson’s *r* was used for all variables (two-tailed tests).

## Discussion

This study presents the results from a clinical study investigating the efficacy of the SMR-BCI with audio-cue, motor observation, and multisensory (robotic, auditory, and visual) feedback compared with conventional therapy for upper limb stroke rehabilitation.

In terms of clinical scale scores (FMA-UE, WMFT, and MBI score), upper limb motor functional improvement was observed in both groups after 2-week intervention. This result is consistent with previous evidence demonstrating the effectiveness of BCI intervention for stroke patients’ UL motor function recovery (Cervera et al., 2018; Wu et al., 2020) and a randomized controlled multicenter trial with post-stroke patients (in subacute and chronic phase), which showed significant improvements of FMA-UE scores in BCI and control group (Frolov et al., 2017). The clinical scale scores of the BG had a significantly greater motor function improvement than the CG at the follow-up week rather than Week 2. Similarly, the percentage of patients reaching MCID and MDC in the BG was greater than that in the CG at Week 4 instead of Week 2. This phenomenon may reflect the effectiveness of the long-term clinical effects of SMR-BCI intervention for post-stroke patients (Wu et al., 2020). BCI training added to conventional therapy may enhance motor functioning of the upper extremity and brain function recovery in patients after a stroke (Kruse et al., 2020). Compared to conventional interventions, we suggest BCI-based training for motor recovery of the upper limbs in patients with stroke (Nojima et al., 2021).

Mu rhythms are suppressed, and their power is attenuated, when engaging in motor activity (Gastaut, 1952), observing actions executed by someone else (Muthukumaraswamy et al., 2004), or imagining performing an action (Pfurtscheller et al., 1996, 2006a,^2010^). Thus, researchers usually link the mu suppression of motor cortex to the motor-relevant brain activation (Pineda, 2006). In this study, no significant difference in mu suppression value was found after 2-week BCI intervention. However, there was a positive trend between functional motor scores (FMA-UE, WMFT, and MBI) with mu suppression of bilateral hemisphere. This positive trend agrees with Bundy et al.’s (2017) study that suggests bilateral cortex activation, especially unaffected cortex activation, can contribute to the motor-relevant task of subacute stroke patients (Calautti et al., 2001). Neuronal reorganization may occur on both the ipsilesional and contralesional hemispheres during recovery to regain motor function and therefore bilateral activation for the hemiparetic side is often observed (Dodd et al., 2017). Meanwhile, this activation pattern gives a possible method to control the SMR-BCI system for stroke rehabilitation reliably via the bilateral cortex EEG-based information fusion (Bundy et al., 2017). Therefore, the unaffected hemisphere may play a role in motor recovery following stroke (Gould et al., 2021). However, there is an opinion that increased activation in the intact hemisphere may hinder reorganization in the lesioned hemisphere, which may have a negative impact on recovery. In response to the two contradictory views, a bimodal balance-recovery model that links interhemispheric balancing and functional recovery to the structural reverse was suggested (Di Pino et al., 2014). As our previous work has shown that motor function recovery and the brain networks of stroke patients could be improved significantly by 4-week SMR-BCI intervention. We conjecture that this bilateral activation, as well as the positive correlations between mu suppression and clinical scores, is a “middle state” for the patients in this study and may also mean that a 2-week training period would not be the optimal option for neurorehabilitation. This result is consistent with the conclusion that a BCI training with conventional therapy for a duration of 4 weeks or longer, with a high intensity training of five times per week, was recommend (Kruse et al., 2020). The influence of the training duration combined with the training intensity needs to be investigated further.

Several features distinguish this work from previous studies. Firstly, we developed a new SMR-BCI system for stroke rehabilitation. This BCI system showed a more vivid training experience for patients via audio-cue, motor observation, and multisensory (robotic, auditory, and visual) feedback to make subjects deeply involved in the training. Secondly, this study revealed the clinical efficacy of the SMR-BCI system we designed for post-stroke rehabilitation. Thirdly, the prescription (e.g., the intensity/frequency and the duration) of BCI training was discussed. We provided a training program (high training intensity, short training duration, and 1-h training session), which may help to develop a full picture of the clinical factor and the interaction between training duration and training intensity.

There were also several limitations to note. The small sample of participants and the absence of EEG data from the CG and follow-up week limited the further investigation of our findings on efficacy. Although the influence of patients’ age and type of lesion in motor recovery of upper extremities of post-stroke patients need further analysis (Kruse et al., 2020), there are some views that relatively younger and more hemorrhagic stroke participants in the BG may tip off the outcome measures comparison between the groups.

## Conclusion

In summary, this study showed the clinical efficacy of SMR-BCI with audio-cue, MO, and multisensory (robotic, auditory, and visual) feedback for post-stroke rehabilitation. Moreover, it discussed the impact of the high training intensity BCI training with short training duration.

Clinical efficacy was measured by three clinical measure scales (FMA-UE, WMFT, and MBI), and the results showed significant improvements at Week 2 and Week 4. Notably, there was a greater improvement for patients who belonged to the BG than the CG at Week 4.

Hence, in the future, more extensive clinical trials are warranted to verify the clinical efficacy and the role of this kind of BCI system has in the rehabilitation milieu. The discussion about the interaction between training intensity and duration also motivates further research.

## Data Availability Statement

The raw data supporting the conclusions of this article will be made available by the authors, without undue reservation.

## Ethics Statement

The studies involving human participants were reviewed and approved by The Third Affiliated Hospital of Sun Yat-sen University, Guangzhou, China. The patients/participants provided their written informed consent to participate in this study.

## Author Contributions

XL, LW, SM, ZY, ZT, LS, YZ, XW, SW, JW, and ZD worked together to complete the manuscript. XL, LW, SW, JW, and ZD contributed to conception and design of the study. LW and SM carried out the data analysis. LS carried out the experiments. ZY and ZT provided statistical assistance and support. YZ and XW provided opinions on grammar and rhetoric. All authors contributed to manuscript revision, read and approved the submitted version.

## Conflict of Interest

The authors declare that the research was conducted in the absence of any commercial or financial relationships that could be construed as a potential conflict of interest.

## Publisher’s Note

All claims expressed in this article are solely those of the authors and do not necessarily represent those of their affiliated organizations, or those of the publisher, the editors and the reviewers. Any product that may be evaluated in this article, or claim that may be made by its manufacturer, is not guaranteed or endorsed by the publisher.
